# Ontogenetic changes in skeletal muscle fiber type, fiber diameter and myoglobin concentration in the Northern elephant seal (*Mirounga angustirostris*)

**DOI:** 10.3389/fphys.2014.00217

**Published:** 2014-06-10

**Authors:** Colby D. Moore, Daniel E. Crocker, Andreas Fahlman, Michael J. Moore, Darryn S. Willoughby, Kathleen A. Robbins, Shane B. Kanatous, Stephen J. Trumble

**Affiliations:** ^1^Department of Biology, Baylor UniversityWaco, TX, USA; ^2^Department of Biology, Sonoma State UniversityRohnert Park, CA, USA; ^3^Department of Life Sciences, Texas A&M UniversityCorpus Christi, TX, USA; ^4^Department of Biology, Woods Hole Oceanographic InstitutionWoods Hole, MA, USA; ^5^Department of Health, Human Performance and Recreation, Baylor UniversityWaco, TX, USA; ^6^Department of Biology, College of Natural Sciences, Colorado State UniversityFort Collins, CO, USA

**Keywords:** elephant seal, fiber typing, myoglobin, diving, ischemia reperfusion injury

## Abstract

Northern elephant seals (*Mirounga angustirostris*) (NES) are known to be deep, long-duration divers and to sustain long-repeated patterns of breath-hold, or apnea. Some phocid dives remain within the bounds of aerobic metabolism, accompanied by physiological responses inducing lung compression, bradycardia, and peripheral vasoconstriction. Current data suggest an absence of type IIb fibers in pinniped locomotory musculature. To date, no fiber type data exist for NES, a consummate deep diver. In this study, NES were biopsied in the wild. Ontogenetic changes in skeletal muscle were revealed through succinate dehydrogenase (SDH) based fiber typing. Results indicated a predominance of uniformly shaped, large type I fibers and elevated myoglobin (Mb) concentrations in the longissimus dorsi (LD) muscle of adults. No type II muscle fibers were detected in any adult sampled. This was in contrast to the juvenile animals that demonstrated type II myosin in Western Blot analysis, indicative of an ontogenetic change in skeletal muscle with maturation. These data support previous hypotheses that the absence of type II fibers indicates reliance on aerobic metabolism during dives, as well as a depressed metabolic rate and low energy locomotion. We also suggest that the lack of type IIb fibers (adults) may provide a protection against ischemia reperfusion (IR) injury in vasoconstricted peripheral skeletal muscle.

## Introduction

Kooyman et al. (1980) defined the aerobic dive limit (ADL) as the maximum dive duration maintained with aerobic metabolism, while studying Weddell seals (*Leptonychotes weddellii*). These authors discovered that this pinniped species maintains oxygen stores in relation to metabolic rate (oxygen consumption) during diving. This maintenance of aerobic metabolism during breath-hold diving is accompanied by a reduction in cardiac output, vasoconstriction and lung collapse (Scholander, 1940; Kooyman, 1973; Kooyman et al., 1981). Deep diving pinniped species also display physiological adaptations of their locomotor muscle that lengthen ADL, including elevated concentrations of Mb and aerobic enzymes, reliance on lipids for fuel and a prevalence of type I, slow twitch, oxidative muscle fibers (Lenfant et al., 1970; Hochachka and Foreman, 1993; Kanatous et al., 1999, 2002; Trumble and Kanatous, 2012). These adaptations, as well as energy-saving swimming behavior (Kooyman et al., 1980; Williams et al., 2000) contribute to extending ADL (Kooyman et al., 1981; Davis and Kanatous, 1999).

The focus of this study was to assess Mb concentrations, fiber type composition, and muscle fiber diameter and cross-sectional area of a primary locomotor muscle, the longissimus dorsi, in three age classes (pup, juvenile and adult) of NES. Terrestrial species, such as mice have comparatively smaller diameter oxidative (type I) fibers, which are interspersed between larger, anaerobic fibers (type II) in a heterogeneous fiber population, such that type I (slow twitch oxidative), type IIa (fast twitch oxidative-glycolytic) and IIb (fast-twitch glycolytic) are present in fiber bundles, and were considered during this study (Scott et al., 2001). Comparatively, a deep-diving pinniped such as the Weddell seal, appear to have a skeletal muscle composition containing only large type I fibers (Kanatous et al., 2002; Trumble et al., 2010). Alternatively, California sea lions (CSL) (*Zalophus californianus*) are relatively shallow divers (Feldkamp et al., 1989; Weise et al., 2006) and have a more heterogeneous fiber type distribution in the muscle given different dive durations and rates of muscular activity, all of which could determine recruitment of glycolytic fibers (Ponganis and Pierce, 1978).

Myoglobin concentrations also play a key role in the metabolic profile of marine mammals (Dearolf et al., 2000; Kanatous et al., 2002; Watson et al., 2003; Williams and Noren, 2011; Trumble and Kanatous, 2012; Kielhorn et al., 2013; Velten et al., 2013). As the oxygen binding protein in skeletal muscle, Mb controls the release and utilization of oxygen during hypoxia (Salathe and Chen, 1993), and elevated concentrations are correlated with increased breath-holding times (Kooyman, 1989; Noren and Williams, 2000). Both diving mammals and diving birds demonstrate elevated levels of Mb (Scholander, 1940; Castellini and Somero, 1981; Kooyman, 1989). For example, the NES, which is capable of repeated dives to mean depths of 516 ± 53.2 m for 23.1 ± 2.6 min (Le Boeuf et al., 2000; Robinson et al., 2012), have skeletal muscle Mb concentrations (7.5 ± 0.7 g/100 g) among the highest reported in mammals (Hassrick et al., 2010). Previous research on Mb and associated muscle enzyme concentrations of the NES (Thorson and LeBoeuf, 1994), Weddell seal (Kanatous et al., 2008; Trumble et al., 2010), harp seal (*Pagophilus groenlandicus*), hooded seal (*Cystophora cristata*) (Lestyk et al., 2009), harbor seal (*Phoca vitulina*) (Jørgensen et al., 2001) and gray seal (*Halichoerus grypus*) (Noren et al., 2005) has revealed that muscle development proceeds gradually during ontogeny (Kanatous et al., 1999; Picard et al., 2002; Noren et al., 2005; Burns et al., 2007). Specifically, blood-based oxygen storage calculations and measurement of muscle-based Mb in the NES during the10-week period post weaning, demonstrate an increase in Mb concentrations from 2.1 g/100 g tissue in weaners to 5.7 g/100 g tissue in juveniles (Thorson and LeBoeuf, 1994). This correlates with the deep dive depths (206 m) reached by juvenile seals during their first few weeks at sea and indicates ontogeny related changes in Mb expression (Thorson and LeBoeuf, 1994). More recent work has revealed that apnea stimulates the production of Mb protein expression by 60% in elephant seals (Vázquez-Medina et al., 2011) and Mb development may be increased in response to post natal signals such as exercise (Lestyk et al., 2009). Furthermore, it has been suggested that diving Weddell seals have a unique adaptation in the regulation of Mb expression, where hypoxia and lipids prime myocytes for optimal Mb expression (De Miranda et al., 2012). Coupled with muscular activity, elevated Mb concentrations are developed and maintained (De Miranda et al., 2012). The role of lipids as a driving force behind the regulation of Mb expression lends evidence to a correlation between reliance on aerobic lipid-based metabolism and the utilization of oxygen during a dive (De Miranda et al., 2012).

Fiber type distribution has been described in several pinniped species including the grey seal (Reed et al., 1994), harbor seal (Reed et al., 1994; Watson et al., 2003), Antarctic fur seal (*Arctocephalus gazella*) (Reed et al., 1994), and Weddell seal (Kanatous et al., 2002; Trumble et al., 2010). However, knowledge of the ontogeny of diving, as it relates to NES skeletal myofibrillar profile data are limited (Lestyk et al., 2009). To date, skeletal muscle fiber type data are lacking for the NES. It was the aim of this study to determine the differences in the skeletal muscle composition from pup to adult in the NES. We compared fiber type profiles and cross-sectional diameter and area for three age groups (pups, juveniles, and adults) and assessed the changes in Mb concentrations. We hypothesize that NES are adapted for aerobic long-duration exercise by having a predominately type I muscle fiber profile and elevated Mb levels.

## Material and methods

### Specimen collection

We obtained muscle biopsies from 9 adults (1 female and 8 males), 4 juveniles (all males) and 8 recently weaned pups (3 females and 4 males). In addition, a CSL juvenile (*n* = 1) and adult mouse (*n* = 1) were sampled as comparison and control. Average approximate mass (kg) ± *SD* for each NES age class and one CSL are reported in Table 1.

**Table 1:** **Elephant seal average approximate weight (kg) ± *SD* for three age classes and one CSL average weight (kg) ± *SD*, *N* = 22**.

**Species**	**Age class**	**Average weight (kg) ± *SD***
NES	Weaned pup	132 ± 11
NES	Juvenile	167 ± 28
NES	Adult	1385 ± 511
CSL	Juvenile	120

NES were sampled on Año Nuevo State Reserve, California during the breeding and molt haulouts for adults and fall haulout for juveniles in 2012 and 2013. Seals were anesthetized using an intramuscular injection of Telazol (teletamine/zolazepam HCl) at a dose of 1.0 mg/kg and intravenously administered doses of ketamine and diazepam (as needed) to maintain immobilization (Fort Dodge Laboratories, Ft. Dodge, IA) (Crocker et al., 2012). To access the LD, a 2 cm^2^ area was cleaned with betadine before each incision (2 cm). For standardization purposes, samples were taken from the mid-belly of the muscle and at the same location in all age classes (one-third of the body length from the tip of the tail). Muscle biopsies (connective tissue and blood dissected away) were collected (30–50 mg) and taken under local anesthetic (1 ml; Lidocaine®, Whitehouse Station, NJ, USA) using a 6 mm biopsy cannula (Depuy, Warsaw, IN, USA). Muscle samples were stored in a liquid nitrogen dry vapor shipper (Thermo Scientific) until long-term storage in a −80°C freezer. Specimens were collected under NMFS marine mammal permit #14636 and all procedures were approved by the Sonoma State University IACUC. LD muscle samples were specifically chosen, as LD is the major locomotory muscle in NES. However, biopsies may represent a small subset of muscle from a large animal and can be considered a limitation to this study. Two other muscles were collected to act as control for the methods described. The locomotory muscle (pectoralis major) of the CSL (CSL10281), housed at The Marine Mammal Center in Sausalito, California was sampled immediately following death. For a terrestrial mammal comparison, the hindlimb locomotor muscle (biceps femoris) from a mouse was harvested (Jackson Laboratory in Bar Harbor, ME). NES samples were sent frozen in cryovials and kept in a −80°C freezer approximately one month prior to analysis.

### Muscle fiber typing, diameter, and cross-sectional area

Muscle bundles were oriented to ensure that the long axis of the isolated myofibers were perpendicular to the cryostat blade. Cross-sections were sliced frozen (−20°C) at 10 μm using a cryostat (Bright Instrument Co., OTF). Serial sections were placed on glass slides and stained for SDH according to the methods of Dearolf et al. (2000). These methods have been successfully used in previous marine mammal fiber typing analyses (Watson et al., 2003; Cotten et al., 2008; Kielhorn et al., 2013; Velten et al., 2013).

Slides were incubated in a 0.2 M sodium phosphate buffer solution, sodium succinate (13.025 g/250 ml) and nitro blue tetrazolium chloride (NBT; 0.015 g/30 ml). NBT, a purple-colored salt, binds to the electron acceptor following the oxidation of succinate, resulting in a purple staining pattern within the mitochondria of each muscle cell, and thus offers a good marker for mitochondrial abundance in muscle fibers (DiMauro et al., 2012). Incubation time was approximately 60 min at 37°C, followed by a 2 min rinse in saline solution (1.96 g/200 ml), 10 min fixation step in formalin-saline solution (10 ml/90 ml) and a 5 min rinse in 15% ethanol. All slides were not incubated together which may reflect some coloration differences between slides. Stained cross-sections were dried and mounted with cover slips. Samples were analyzed in triplicate using a high-resolution camera-mounted microscope (Nikon Eclipse Ci; Nikon, Brighton MI, USA).

Ten fibers from each fiber bundle were counted and measured based on consistent orientation, where dissected fiber bundles were tightly arranged, round and whole (adapted from Velten et al., 2013). Only fiber types commonly found in mammalian skeletal muscle can be determined by SDH staining methods, thus type I, type IIa, and type IIb were considered during this study. SDH stain designates the relative oxidative potential of each fiber type via a colorimetric method. There is a positive linear correlation between color and oxidative potential. Type I fibers stained darkest, followed by a decreasing color spectrum of type IIa and type IIb. Given the similar staining intensity for all fibers within NES cross-sections, the SDH stained fibers were qualified as one fiber type, where all whole, round fibers were counted. This was in contrast to the terrestrial mouse where fibers were individually qualified based on their fiber type.

Average fiber diameter and area were measured to scale using a high-resolution microscope with accompanying camera (Nikon Eclipse Ci) and software (NIS element D) calibrated at 200× magnification. Data are reported here as mean μm ± standard deviation (SD) for each fiber sampled (Table 2). Freeze fracture was visible in some muscle cross-sections and deemed unavoidable due to field sampling protocol. These fibers were not utilized when assessing fiber size.

**Table 2:** **Elephant seal fiber diameters (**μ**m), average cross-sectional area (**μ**m^2^) per age class (mean ± *SD*, *N* = 11) and average myoglobin (mg/g) concentrations (mean ± *SD*, *N* = 9)**.

**Age class**	**Average fiber diameter**	**Average cross-sectional area**	**Average Mb concentration (mg/g)**
Pup	41.9 ± 5.9 (a)	1406.8 ± 397.4	21.0 ± 7.2 (a)
Juvenile	64.4 ± 12.6 (a)	3373.8 ± 1327.3	51.8 ± 13.7 (b)
Adult	118.7 ± 21.1 (b)	11410.1 ± 3941.8	59.1 ± 4.1 (b)

Letters denote statistical significance.

### Western blotting

Muscle tissue was homogenized using a Bullet Blender (Next Advance, NY USA) in CellLytic MT buffer (Sigma Aldrich) using 0.5 mm Zirconium oxide beads. The supernatant was aliquoted and utilized for Bradford assay (Beckman Coulter DU 730 spectrophotometer at 595 nm) to determine total protein content. Standard Western Blot protocol (Abcam) was performed using 8 ul of sample and ladder (Bio-Rad) loaded into SDS gel wells (ClearPAGE; 4–20%), run in a Tris-Tricine SDS running buffer (ClearPage) and transferred using Tris/Glycine buffer (Bio-Rad). Three primary antibodies specific to the myosin heavy chains I, IIa and IIb (Developmental Studies Hybridoma Bank, University of Iowa, BA-D5 (1:750), SC-71 (1:500) and BF-F3 (1:1) respectively) and two secondary antibodies [KPL peroxidase labeled goat anti-mouse IgG (H+L) at 1:10,000, Invitrogen HRP goat anti-mouse IgG+A+M at 1:30,000] were used. Primary antibodies have been previously confirmed for fiber typing in pinnipeds (Watson et al., 2003; Kanatous et al., 2008). Protein bands on a nitrocellulous membrane were visualized using a chemiluminescent substrate kit (KPL International) sensitive to peroxidase-labeled antibodies and developed using a luminescent image analyzer (GE LAS 4000) and accompanying software (GE Healthcare Life Sciences). Western Blot analysis was completed in duplicate.

### Myoglobin quantification

Mb assays were completed using methods modified by Kanatous et al. (1999) from Reynafarje (1963). Homogenates (prepared as described above) were diluted in phosphate buffer (0.4 M KPO4 at pH 6.6) and centrifuged at 28,000 g for 50 min. Supernatant was bubbled with carbon monoxide for 3 min and spectrophotometric absorbance was determined. Absorbance was measured in triplicate at two wavelengths and extinction coefficients (14.7 × 10^3^ cm^−1^M^−1^ at 538 nm and 11.8 × 10^3^ cm^−1^M^−1^ at 568 nm). Mb concentration was calculated and reported as mg/g of muscle mass in Table 2.

### Statistical analysis

Data were analyzed and log transformed for normalization (homogeneity of variances was determined using the Brown-Forsythe test) and statistical significance was maintained at or below the 0.05 alpha level for analysis of variance (ANOVA) and Tukey-Kramer HSD testing. Results are presented here as means ± standard deviation (SD).

## Results

### Muscle fiber type distribution and diameter

**Figure 1:**
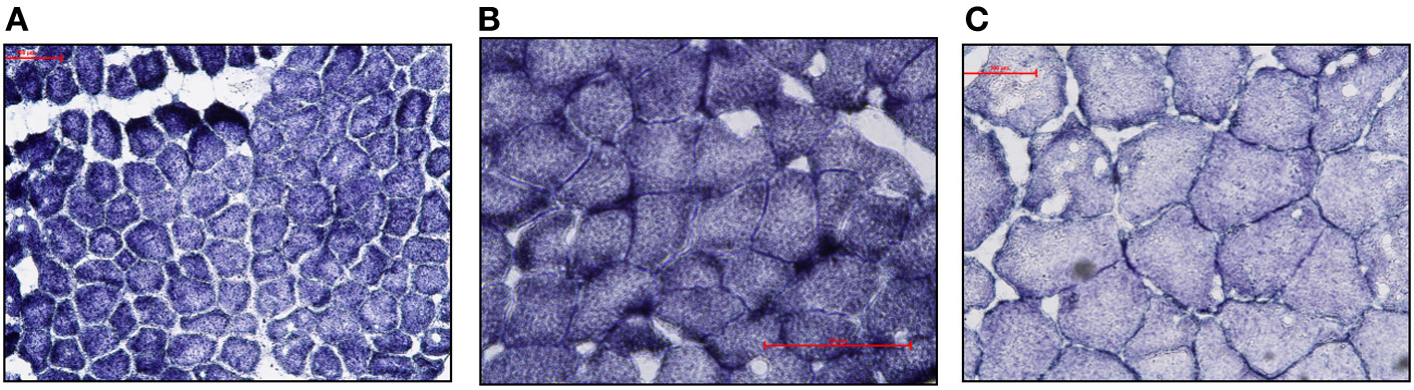
**(A–C)** Photomicrographs (100 μm scale) of cross-sections of Northern elephant seal *longissimus dorsi* muscle stained with succinate dehydrogenase. The succinate dehydrogenase stain is known to correlate with muscle fiber type (Dearolf et al., 2000). Samples from pup **(A)** (6606), juvenile **(B)** (Ele#4), and adult **(C)** (3TC) are shown. Fibers uniformly demonstrate the same staining intensity and cross-sectional diameter within individuals indicative of the presence of a single fiber type.

**Figure 2:**
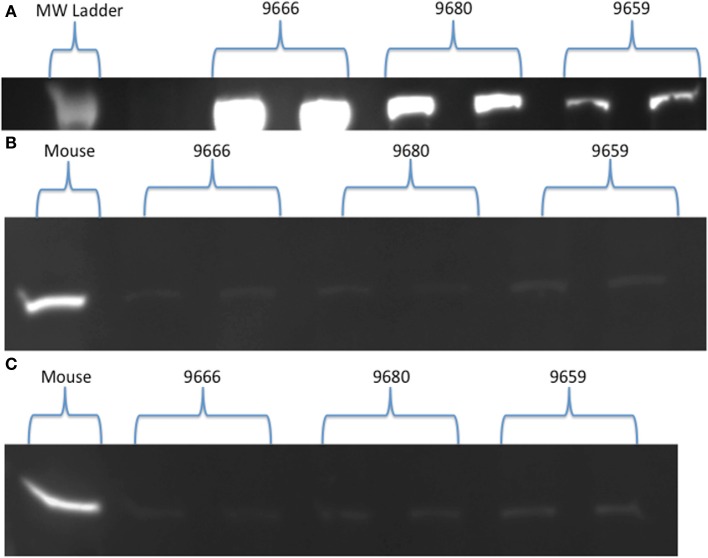
**(A–C)** Western Blot results for Northern elephant seal pups (9666, 9680, 9659). Type I **(A)**, type IIa **(B)**, and type IIb **(C)** skeletal muscle myosin heavy chain antibodies are present in duplicate wells, with either a molecular weight (MW) ladder **(A)** or mouse control **(B,C)** shown for comparison. Qualitative comparisons can be seen between the bright type I **(A)** bands vs. the light type IIa **(B)** and IIb **(C)** bands, indicative of the presence of all myosin types in pup longissimus dorsi muscle, where type I strongly binds and type IIa and IIb slightly bind.

**Figure 3:**
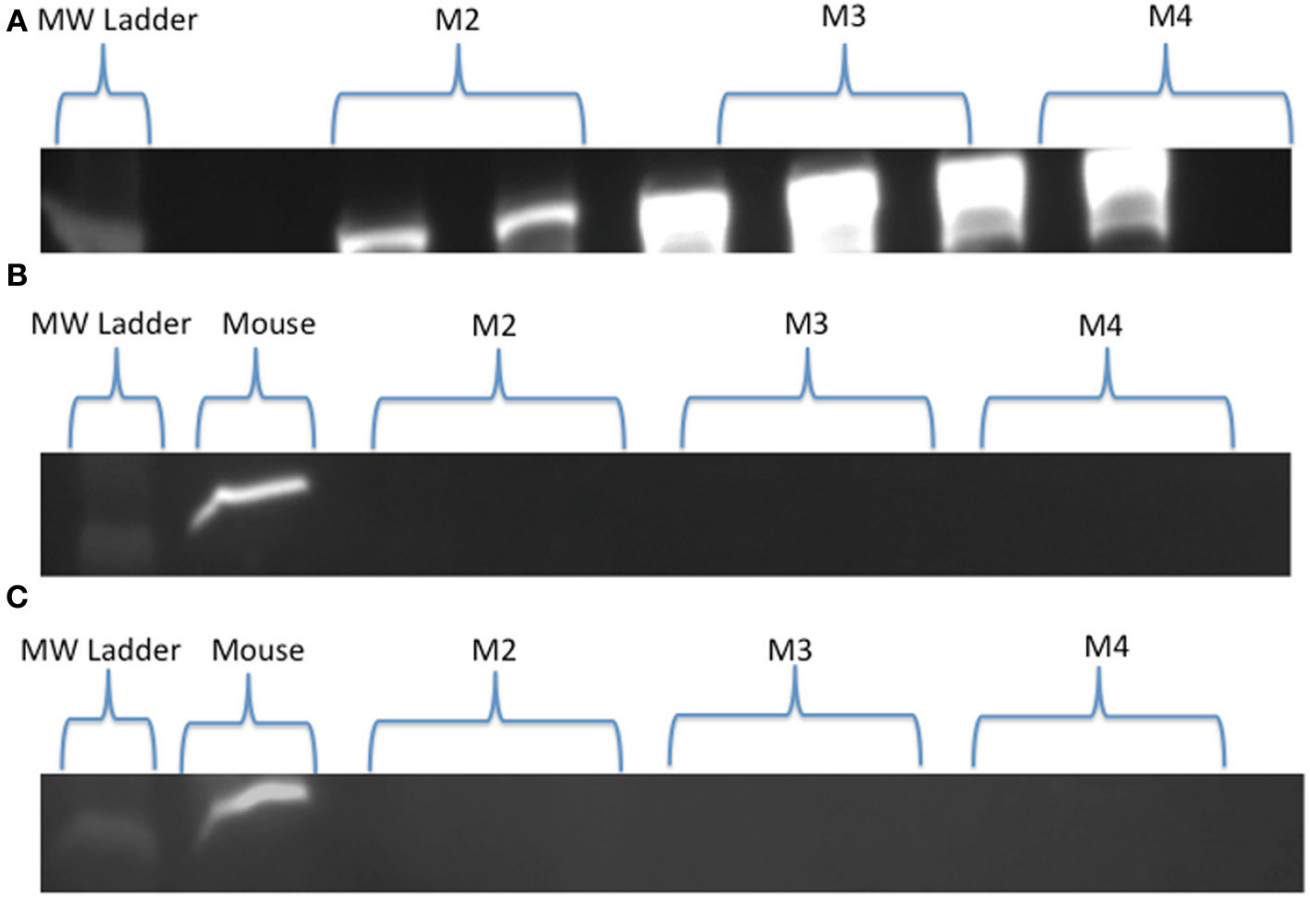
**(A–C)** Western Blot results for Northern elephant seal adults (M2, M3, M4). Type I **(A)**, type IIa **(B)**, and type IIb **(C)** skeletal muscle myosin heavy chain antibodies are present in duplicate wells, with either a molecular weight (MW) ladder **(A)** or mouse control **(B,C)** shown for comparison. Qualitative comparison, specifically absence vs. presence, can be seen between the present bright bands of the type I **(A)** vs. the absent bands of the type IIa **(B)** and IIb **(C)** myosin of the adults, indicating the presence of one fiber type (type I) in adult longissimus dorsi muscle.

Visual examination of SDH stained cross-sections of LD myofibers for all age classes of NES revealed only one type of fiber (Figure 1). Based on Western Blot analysis, the predominate fiber type was type I, where pups (*n* = 3) had slight antibody specific binding to both type IIa and IIb myosin (Figure 2) and adults (*n* = 6) did not demonstrate any binding to type IIa and IIb antibodies, just type I (Figure 3). Therefore, our analysis indicates that myosin fiber type changes over the maturation of the NES. Unlike the LD of the NES, pectoralis muscle of the CSL possessed three different muscle fiber types as did the mouse biceps femoris (supplementary data).

**Figure 4:**
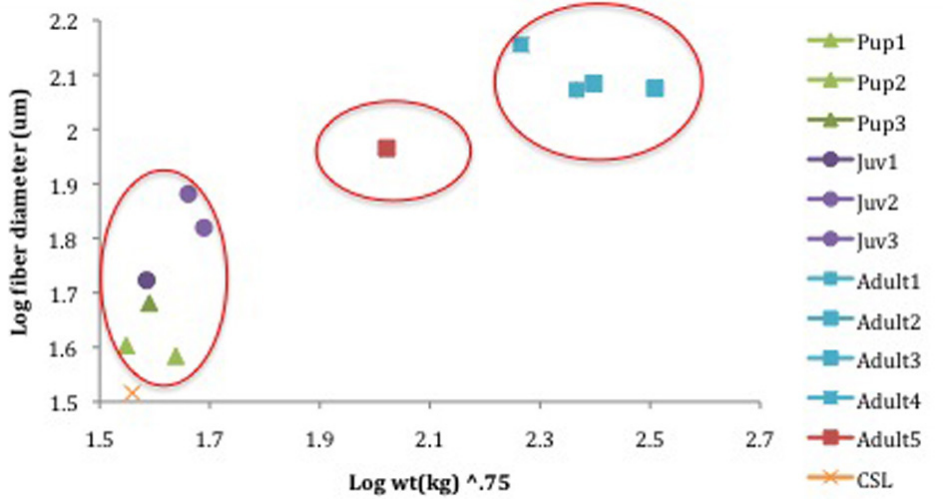
**Fiber diameter (um) in correlation with increasing scaled animal mass (kg^.75^).** Pups (7515, 7606, 6606) = *M. angustirostris*, juv (ele4, FJ11, FJ13) = *M. angustirostris*, adult 1–4 all male (Male2, 6th, 3TC, 7728M08) = *M. angustirostris*, adult 5 only female (FemaleTrip2), CSL = *Z. californianus*. Red circles indicate large groups of similar animals, from top right: adult male, adult female, pup, and juveniles. The California sea lion scales smaller than all Northern elephant seals and is not encircled.

There was a statistical difference in fiber diameters for NES pups (*n* = 4), juveniles (*n* = 4) and adults (*n* = 5) among age classes (pups, juveniles < adults; ANOVA; *p* < 0.05, Table 2) but not within individuals of each age class (ANOVA; *p* > 0.05, Table 2). Fiber diameters were used to calculate average fiber cross-sectional area for each age class of NES. Pups had an average of 1406.8 ± 397.4 μm^2^. Juvenile animals had an average of 3373.8 ± 1327.3 μm^2^ with adults averaging 11410.1 ± 3941.8 μm^2^. The fiber cross-sectional area was also representative of the increase in fiber size with age (Figure 4, Table 2).

Fiber size also varied across species and fiber types (Table 3). Adult NES had relatively large LD fibers throughout cross-sections (Table 2). For both the control mouse and CSL, type I fibers had the smallest mean diameter and type IIb the largest mean diameter (Table 3). Fiber diameters for type I, IIa and IIb (within each animal) were significantly different (ANOVA; *p* < 0.05, Table 3). The NES had a uniformly sized fiber type population in the LD muscle among each age class (Figure 1; Table 2).

**Table 3:** **Fiber diameters (**μ**m) for mouse and California sea lion (mean ± *SD*, *N* = 2)**.

**Animal**	**Type I**	**Type IIa**	**Type IIb**
Mouse	35.1 ± 4.8	48.4 ± 7.5	59.9 ± 7.3
California sea lion	32.8 ± 5.3	42.5 ± 6.2	54.6 ± 6.7

### Myoglobin concentrations

**Figure 5:**
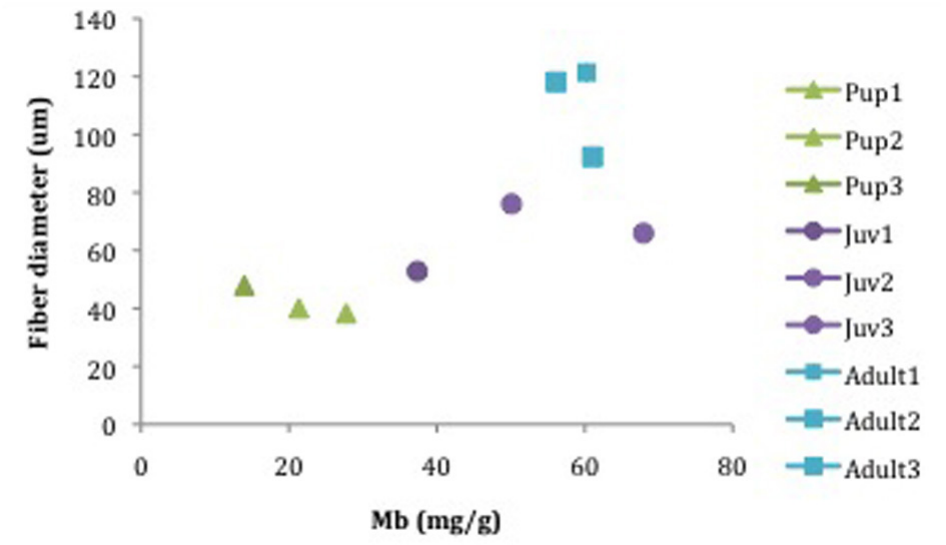
**Myoglobin concentration (mg/g) in correlation with fiber diameter (um) in three age classes of the Northern elephant seal: Pups (7515, 7606, 6606), Juv (ele#4, FJ13, FJ11), and Adults (6th, Male2, FemaleTrip2)**.

NES pups (*n* = 3) had the lowest average concentration of Mb in LD musculature, averaging 21.0 ± 7.2 mg/g of protein while juveniles (*n* = 3) had a concentration of 51.8 ± 13.7 mg/g [Figure 5, Table 2; (*p* < 0.05)]. Adult (*n* = 3) elephant seals had the highest average Mb concentration of 59.1 ± 4.1 mg/g of protein (Figure 5, Table 2; pup < juvenile, adult, Tukey-Kramer HSD, *p* < 0.05). Mb concentrations were calculated for NES age classes only; CSL and mouse Mb concentration was not determination due to sample size constraints. Mb concentration was compared to the fiber diameter, and indicated the positive correlation of Mb concentration with increasing fiber diameter (Figure 5).

## Discussion

This is the first study to describe muscle fiber profile changes across ontogeny in NES skeletal muscle. Here, we determined that the adult NES, a deep-diving phocid (Le Boeuf et al., 2000; Kuhn et al., 2009; Robinson et al., 2012), has uniformly large and metabolically uniform (SDH) type I fibers in the fiber bundles of the LD muscle investigated in this study. Western Blot analysis revealed pup muscle has binding for type IIa and IIb myosin heavy chain, demonstrating ontogenetic changes in fiber type. In conjunction, we show age-related increases in Mb concentration, where Mb is positively correlated to fiber diameter.

### Ontogenetic change in fiber type and large muscle fibers

Western Blot analysis demonstrated a distinct fiber population in immature seals as compared to adult seals. Pups expressed myosin I antigens, and to a smaller extent IIa and IIb antigens, whereas adult muscle expressed solely type I antigens (Figures 2, 3). The absence of type II fibers in adults in this study was similar to previous studies on harbor seal and Weddell seal locomotory musculature, where one or both anaerobic fibers are absent (Kanatous et al., 2002; Watson et al., 2003; Trumble et al., 2010). In contrast, the CSL (supplementary data) as well as some cetaceans have type IIa and type IIb fibers in locomotory musculature (Kielhorn et al., 2013; Velten et al., 2013), indicating different models for exercise. Low oxidative capacity (SDH color intensity) was observed in association with the type I fibers in adults. This result has also been seen in Weddell seals (Kanatous et al., 2008), and could be indicative of low mitochondrial densities within myofibers and a low rate of oxygen consumption (Kanatous et al., 2002).

The presence of type II myosin in pups, but absence in adults, indicates that muscle plasticity, or shifts in muscle myosin type, occur with age in NES. Previous research on Weddell seals confirms the existence of a juvenile fiber profile (Trumble et al., 2010), suggesting an element of dive training and shift in muscle myosin with age. Muscle fiber plasticity has been documented in mammals, and the change in contraction speed and metabolic basis (fiber type) is thought to occur in response to various stimuli (Pette and Staron, 1997; Grossman et al., 1998; Ricoy et al., 1998; Scott et al., 2001). Neonatal dolphins were found to have different mitochondria and lipid content than adults, indicative of a lower aerobic capacity (Dearolf et al., 2000) and demonstrating marine mammal ontogenetic changes in musculature. Fiber conversion, specifically, has been seen between type IIa and IIb with type I to II also possible (Pette and Staron, 1997; Scott et al., 2001). Less common is the shift from type II to type I (Scott et al., 2001), although recent data show the activation of certain muscle-specific proteins can generate an “endurance athlete” mouse model with increased levels of aerobic enzymes, mitochondria and type I fibers (Wang et al., 2004). This would indicate that muscle plasticity, specifically transformation to type I could be promoted with endurance training. Muscle fiber type conversion in development for NES indicates that muscle cells in young animals are also plastic and muscle type may be dependent on nerve activity (Eken and Gundersen, 1988). In a more general sense, exercise stimuli based on extensive deep diving as a juvenile after/during the first trip to sea might stimulate muscle fiber type plasticity. Similarly, Weddell seals also demonstrate muscle plasticity when exposed to muscular activity and hypoxia (De Miranda et al., 2012). Perhaps the expression of anaerobic antigens can be considered an intermediary developmental link between age groups, where anaerobic antigens are not present in the adult group when an optimal concentration of Mb is achieved. Thus, smaller diameter fibers of mixed composition are indicative of a more terrestrial dwelling pup and larger homogeneous fibers are indicative of a swimming adult. The adult elephant seal has high concentrations of Mb and relatively large oxidative fibers, with little necessity for anaerobic metabolism, a metabolic state attained through endurance training. Future work could be aimed at quantifying the changes in neural innervations stimulating fiber conversions and how this activity correlates with age and exercise training. Regardless, more than one qualifying/quantifying protocol should be used for fiber determination in diving mammals (Watson et al., 2003).

As a general rule, there is a positive correlation between muscle fiber diameter and oxygen diffusion distance in mammals (Van Der Laarse et al., 1998; Van Wessel et al., 2010). The limitation of having larger fiber diameters is a function of the diffusion distance across the cellular membrane and cell, with smaller fibers allowing for more rapid oxygen diffusion (Van Wessel et al., 2010; Kinsey et al., 2011; Kielhorn et al., 2013). The typically larger anaerobic type II fibers are not constrained by oxygen diffusion distance (Van Wessel et al., 2010; Kielhorn et al., 2013). This was evident in our control mouse (supplementary data) as well as the CSL pectoral muscle (supplementary data), where type I oxidative fibers were significantly smaller than both type IIa and IIb fibers (Table 3). NES had greater mean muscle fiber diameter regardless of age class than the CSL and the mouse control (*p* < 0.05, Tables 2, 3) as well as previously sampled cetaceans and pinnipeds (86.2 μm *K. breviceps*; 66.8 μm *G. macrorhynchus*; 90.5 μm M. europaeus; 94 μm *L. weddellii*) (Kanatous et al., 2002; Kielhorn et al., 2013; Velten et al., 2013). Previous myofiber studies in the beaked whale (*Mesoplodon* sp.), short finned pilot whale (*Globicephala macrorhynchus*) (Velten et al., 2013), pygmy sperm whale (*Kogia breviceps*) (Kielhorn et al., 2013) and the Weddell seal (Kanatous et al., 2002), report larger relative fiber diameters, suggesting an oxygen conserving adaptation for diving. According to the “optimal fiber size hypothesis” (Johnston et al., 2004; Jimenez et al., 2011), Velten et al. (2013) and Kielhorn et al. (2013) hypothesized that due to low surface area to volume ratios of enlarged muscle fibers and associated dampened metabolic cost for membrane potential, enlarged fibers may result in lower muscular energetics and thus lower metabolic rates (Kielhorn et al., 2013).

### Elevated myoglobin concentrations

In humans, exercise prompts an increased consumption of oxygen that is compensated by increased blood flow (Blomstrand et al., 1997). This is counter to breath-hold diving pinnipeds, known to vasoconstrict and decrease blood flow to peripheral skeletal muscle (Zapol et al., 1979). Therefore, diving pinnipeds must store greater amounts of Mb in musculature as on-board oxygen storage during a dive (Kanatous et al., 1999) indicated by reports of increases in Mb expression after a juvenile's first trip to sea and achievement of deep dive depths (Thorson and LeBoeuf, 1994). In this study, we found increasing Mb levels with age and body mass (Figure 5). Juveniles and adults had significantly higher levels of Mb than pups (Figure 5, Table 2, *p* < 0.05). Mean Mb values reported during this study for the adult NES (59.1 ± 4.1 mg/g) are similar to other pinniped species such as the adult Weddell seal (45.9 ± 3.3 mg/g: Kanatous et al., 2002), harbor seal (38 ± 1 mg/g: Polasek et al., 2006; 37.4 ± 1.7: Kanatous et al., 1999; 41 ± 4: Reed et al., 1994) and Stellar sea lion (28.7 ± 1.5 mg/g: Kanatous et al., 1999). Compared to terrestrial species (domestic dog: 3.5 mg/g, Kanatous et al., 2002), NES and other species noted above have much greater Mb concentrations in their locomotory musculature. Thus, large intraspecific variation exists for Mb concentration as Mb values have been determined to vary with activity level (De Miranda et al., 2012). Noren and Williams (2000) found that diving proficiency is correlated with elevated Mb concentration and increased body mass in some odotocete species. This correlation appears to apply to pinnipeds as well (Figure 5). Similarly, in this study, the correlation of Mb (mg/g tissue) and body mass suggests that a shift occurs during ontogeny, where juvenile animals demonstrate increased levels of Mb (Figure 5). These data are concurrent with other species of diving mammals where Mb expression in skeletal muscle is triggered through exposure to hypoxia (De Miranda et al., 2012).

### Possible connection to ischemia reperfusion injury

Ischemia and subsequent restoration of blood flow to mammalian body tissue can cause harmful effects, cumulatively often termed ischemia reperfusion (IR) injury. A similar process involving vasoconstriction followed by restoration of blood flow progresses during a pinniped dive (Scholander, 1940), yet no injury has been described. In mice, ischemic events lasting as little as 20 min can cause edema in tissue (Carattino et al., 2000). The release of oxygen-free radicals, or reactive oxygen species (ROS), as a result of the reintroduction of oxygenated blood, causes cellular damage (McCord, 1985; Saugstad, 1996), and thus substantial injury (Li and Jackson, 2002). Diving mammals might avoid harmful effects of IR injury through frequent exposure to ischemia (repetitive dives), which may “precondition” muscle and provide protection (Elsner et al., 1998). Seals may also have enhanced scavenging capacity via elevated activity of superoxide dismutase (SOD) in myocardium (Elsner et al., 1998) and glutathione peroxidase (GPx) in muscle (Vázquez-Medina et al., 2006). Although free radicals are present and play a role in IR injury, their role is secondary to the amplification of the initial injury (Chan et al., 2004), contradicting early research suggesting that IR-related tissue necrosis was the result of ROS (Chan et al., 2003). IR injury has now been linked specifically and primarily to the inflammatory response (Austen et al., 2004; Chan et al., 2004; Suber et al., 2007) responsible for destruction of muscle tissue in mice (Chan et al., 2004). The site of IgM and complement protein deposition (inflammatory proteins primarily responsible for mediation of the injury) is on fast-twitch type IIb muscle fibers (Chan et al., 2004)—absent in adult NES skeletal muscle. Due to the lack of type IIb fibers, deep-diving pinnipeds must also lack the unique expression of epitopes on type IIb fibers, singularly found to bind to immune proteins during the process of injury. We believe that this apparent lack of type IIb fibers in adult locomotory musculature may preclude IR injury in diving pinnipeds. This would suggest that diving cetaceans and even other pinniped species (CSL) represent a different muscular model for deep diving, as they possess type IIb fibers in skeletal muscle (Kielhorn et al., 2013; Velten et al., 2013). A deletion or alteration to the activating immune pathway of the disease is possible, making the presence of type IIb fibers inconsequential. In addition, extent of ischemia in musculature (Meir et al., 2009; McDonald and Ponganis, 2013) may vary and should not be assumed for all marine mammals, as early experiments did not encompass all species (Scholander, 1940), which may cover a wide range of exercise preferences. Future research could also be aimed toward the culture of cells to identify the expression of epitopes or inhibitory proteins expressed in muscle tissue after an ischemic event, especially for animals with type IIb fibers (CSL) which also undergo ischemic insult to muscle tissue during a dive (McDonald and Ponganis, 2013).

In summary, the cross-sectional fiber type profile of the NES LD muscle shown in this study was complementary to what was previously known about mammalian divers. Previous research on the Weddell seal (Kooyman, 1981; Castellini et al., 1992; Davis et al., 1999; Kanatous et al., 2002) revealed a large predominance of type I fibers in locomotory musculature in contrast to short-duration divers with mixed fiber profiles that are able to rely on anaerobic metabolism (Kanatous et al., 1999). One benefit to having a type I fiber distribution is fatigue resistance (Van Wessel et al., 2010). Additionally, type I oxidative muscle fibers produce increased levels of antioxidants (nitric oxide), which act as protection against hypoxic insult (Yu et al., 2008). Cumulatively, scaled larger type I muscle fibers would be beneficial for a long-duration diver wholly dependent on efficient utilization of stored oxygen and suppression of harmful effects of hypoxia. We suggest that the NES maintains a similar diving strategy to the Weddell seal, as low-level aerobic metabolism may be the best model for long, deep diving. The scaled enlarged fiber size, predominately type I fiber profile, and elevated Mb content of skeletal muscle in adult NES allows for an excellent model for adaptation to heightened capability of oxygen storage and utilization. Furthermore, these adaptations coupled with the lack of type IIb fibers may allow seals to avoid a deleterious mammalian disease (IR injury) and withstand repeated ischemia to peripheral tissue.

## Author contributions

Colby D. Moore was primary author and completed data analysis. Andreas Fahlman, Michael J. Moore, Darryn S. Willoughby, and Stephen J. Trumble contributed to concept, and method development; Daniel E. Crocker, Kathleen A. Robbins, Shane B. Kanatous, and Stephen J. Trumble collected and handled biopsy and whole muscle samples.

### Conflict of interest statement

The authors declare that the research was conducted in the absence of any commercial or financial relationships that could be construed as a potential conflict of interest.

## Supplementary material

Figure S1 **(A–C)** Succinate dehydrogenase fiber typing profile from a California sea lion pectoral muscle revealing three fiber types: type I **(A)**, IIa **(B)**, and IIb **(C)**, each designated with circles. The succinate dehydrogenase stain demarks each fiber type based on color intensity correlated with oxidative capacity, where type I oxidative fibers stain darkest and type IIb anaerobic fibers are lightest.

Figure S2 **(A–C)** Succinate dehydrogenase fiber typing profile from a mouse revealing three fiber types: type I **(A)**, IIa **(B)**, and IIb **(C)**, each designated with circles. The succinate dehydrogenase stain demarks each fiber type based on color intensity correlated with oxidative capacity, where type I oxidative fibers stain darkest and type IIb anaerobic fibers are lightest.
