# Supplementary material for: Ontogenetic changes in skeletal muscle fiber type, fiber diameter and myoglobin concentration in the Northern elephant seal (Mirounga angustirostris)
Source: Front Physiol. 2014 Jun 10;5:217. doi: 10.3389/fphys.2014.00217 (PMC4050301; doi:10.3389/fphys.2014.00217)
Supplement: Figure S1 — (A–C) Succinate dehydrogenase fiber typing profile from a California sea lion pectoral muscle revealing three fiber types: type I (A), IIa (B), and IIb (C), each designated with circles. The succinate dehydrogenase stain demarks each fiber type based on color intensity correlated with oxidative capacity, where type I oxidative fibers stain darkest and type IIb anaerobic fibers are lightest. [file Presentation1.PDF]

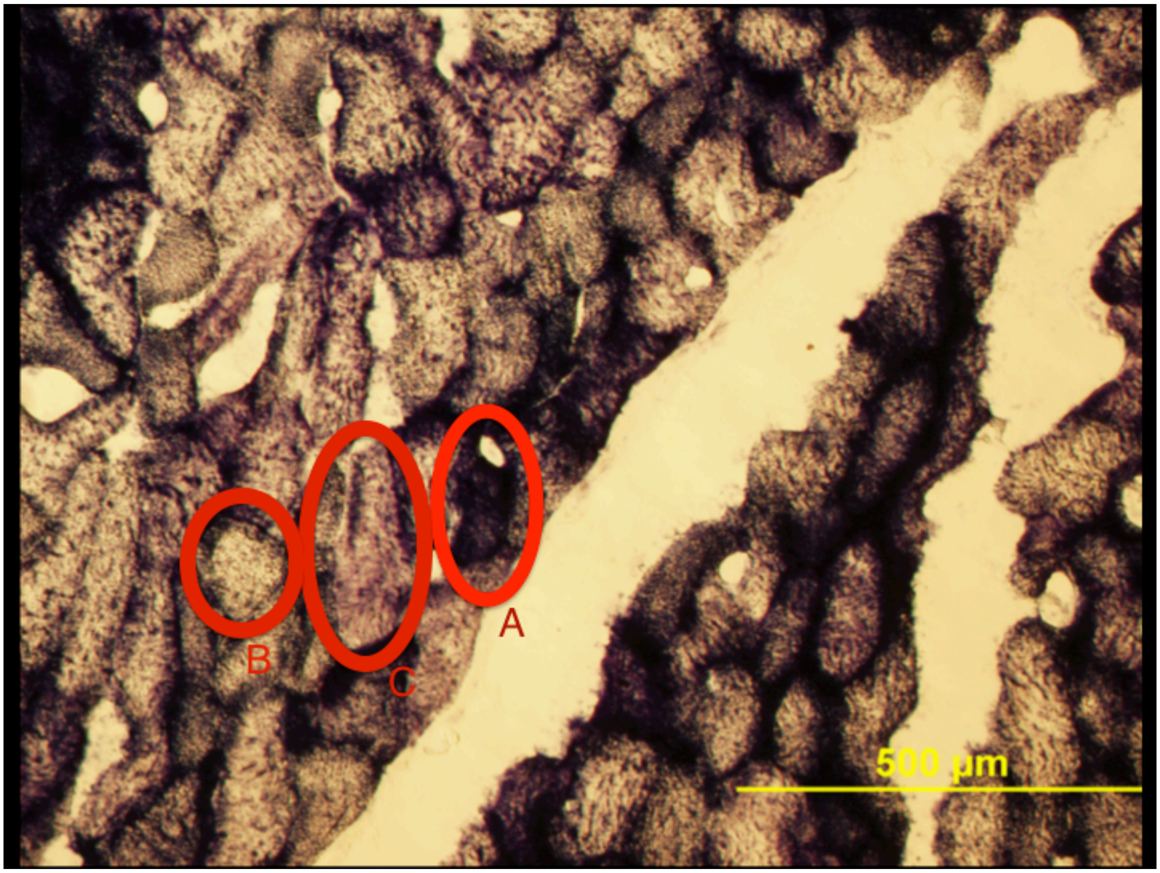

Supplementary figure 1 (**A**-**C**): Succinate dehydrogenase fiber typing profile from a California sea lion pectoral muscle revealing three fiber types: type I (**A**), IIa (**B**) and IIb (**C**), each designated with circles. The succinate dehydrogenase stain demarks each fiber type based on color intensity correlated with oxidative capacity, where type I oxidative fibers stain darkest and type IIb anaerobic fibers are lightest.

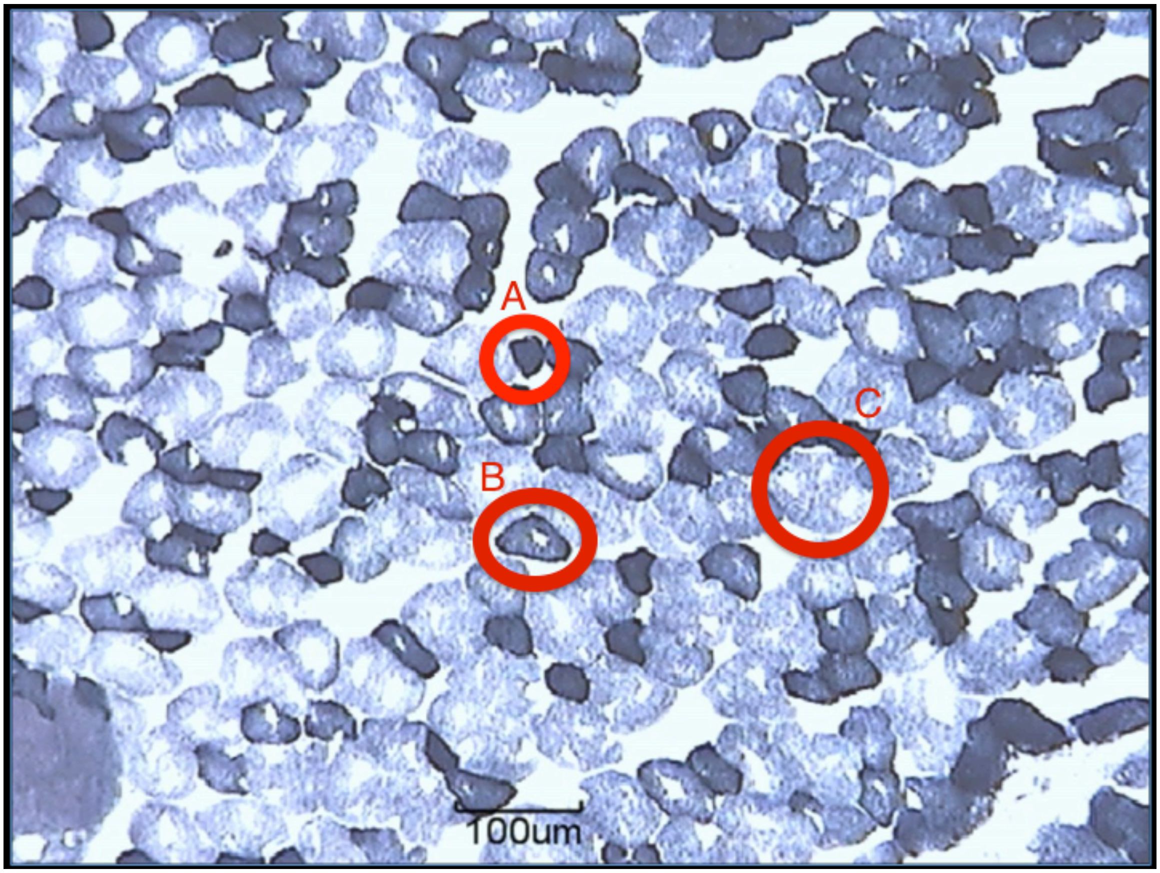

Supplementary figure 2 (**A**-**C**): Succinate dehydrogenase fiber typing profile from a mouse revealing three fiber types: type I (**A**), IIa (**B**) and IIb (**C**), each designated with circles. The succinate dehydrogenase stain demarks each fiber type based on color intensity correlated with oxidative capacity, where type I oxidative fibers stain darkest and type IIb anaerobic fibers are lightest.
